# Stable and Fast-Response Capacitive Humidity Sensors Based on a ZnO Nanopowder/PVP-RGO Multilayer

**DOI:** 10.3390/s17102415

**Published:** 2017-10-23

**Authors:** Hui Yang, Qiangqiang Ye, Ruixue Zeng, Junkai Zhang, Lei Yue, Ming Xu, Zhi-Jun Qiu, Dongping Wu

**Affiliations:** 1 State Key Laboratory of ASIC and System, Fudan University, Shanghai 200433, China; 15110720073@fudan.edu.cn (H.Y.); 14210720116@fudan.edu.cn (Q.Y.); 13110720024@fudan.edu.cn (R.Z.); 15110720077@fudan.edu.cn (J.Z.); 14110720067@fudan.edu.cn (L.Y.); xuming123@263.net (M.X.); 2 School of Information Science and Technology, Fudan University, Shanghai 200433, China

**Keywords:** ZnO nanopowders, RGO, PVP, capacitive humidity sensor

## Abstract

In this paper, capacitive-type humidity sensors were prepared by sequentially drop-coating the aqueous suspensions of zinc oxide (ZnO) nanopowders and polyvinyl pyrrolidone–reduced graphene oxide (PVP-RGO) nanocomposites onto interdigitated electrodes. Significant improvements in both sensitivity and linearity were achieved for the ZnO/PVP-RGO sensors compared with the PVP-RGO/ZnO, PVP-RGO, and ZnO counterparts. Moreover, the produced ZnO/PVP-RGO sensors exhibited rather small hysteresis, fast response-recovery time, and long-term stability. Based on morphological and structural analyses, it can be inferred that the excellent humidity sensing properties of the ZnO/PVP-RGO sensors may be attributed to the high surface-to-volume ratio of the multilayer structure and the supporting roles of the PVP-RGO nanocomposites. The results in this work hence provide adequate guidelines for designing high-performance humidity sensors that make use of the multilayer structure of semiconductor oxide materials and PVP-RGO nanocomposites.

## 1. Introduction

The monitoring and control of environment humidity is very important in various fields, including food, environment, industry, agriculture, and medicine [1,2,3]. Thus far, various methods including resistance [4], impedance [5], capacitance [6], optical fiber [7], field effect transistors (FETs) [8], surface acoustic waves (SAWs) [9], and quartz crystal microbalance (QCM) [10] have been utilized to prepare reliable and low-cost humidity sensors. Capacitance-based humidity sensors exhibited low power consumption, a linear response, and thermal stability [11]. These merits render capacitive-type humidity sensors promising devices for humidity detection. Moreover, several types of humidity sensing materials, such as polymers [12], ceramics [13], metal oxide semiconductors [14], graphene and its derivatives [15,16], have been employed in humidity sensors.

Zinc oxide (ZnO) is one of the most widely applied humidity sensing materials, and it has many merits such as its low-cost preparation, plentiful and controllable surface morphology, perfect chemical and thermal stability, and high electrical sensitivity to humidity [14,17]. Humidity sensors based on ZnO nanostructures with different morphologies, including nanoparticles [18], nanowires [14], nanorods [19], and nanopowders [20], can offer further enhancement in humidity sensitivity due to their unique advantages, including their high specific surface area, their electrochemical activity, and their high conductivity [21]. Among such nanostructures, ZnO nanopowders demonstrate promising sensing properties, including high sensitivity and fast response and recovery times [22]. Therefore, utilizing ZnO nanopowders as the sensing material in capacitive-based structures will be a suitable choice in the development of high-performance humidity sensors. However, humidity sensors based on pure ZnO suffer from disadvantages such as a poor response to low humidity and large humidity hysteresis, which limit its further applications [19,23,24,25]. Researchers have reported on improving the humidity response properties of pure ZnO by doping, surface coating, or mixing with other materials [9,20,26,27,28,29]. However, either the high cost or the comprehensive performance does not meet practical application requirements.

The large specific surface area, high chemical stability, and fascinating electrical properties, such as low noise level and high carrier mobility of reduced graphene oxide (RGO), make it one of the most promising two-dimensional materials for sensing applications [30]. Unfortunately, many researchers have found that RGO itself shows poor sensitivity to relative humidity (RH). This phenomenon was mainly attributed to the low content of oxygen-containing functional groups on the basal edges and planes of RGO [28,31,32]. Considering that RGO is a highly conductive *sp*^2^ carbon atom film that can serve as an anchor to promote electron transfer with other sensing materials [33], it has attracted great attention as a promising candidate for use in humidity sensors. Moreover, RGO is often used as a supporting sensing material for polymers [34,35,36] and semiconducting oxides based sensors [30,33]. Since RGO has poor solubility in water, the compatibility and dispersion of RGO in an aqueous solution is of great concern while preparing thin-film humidity sensors based on RGO. Polyvinyl pyrrolidone (PVP) is easily soluble in water, so it is a suitable organic additive with which the uniform dispersal of RGO can be achieved in an aqueous solution. This method allows for the deposition of regular arrays of polyvinyl pyrrolidone–reduced graphene oxide (PVP-RGO) on a substrate via drop-coating. 

The aim of this work was to investigate capacitive humidity sensors based on PVP-RGO and ZnO nanomaterials for realizing high performance using a simple drop-coating method. It was found that the ZnO/PVP-RGO sensors in the form of a multilayer structure, i.e., a PVP-RGO film superimposed on a ZnO film, exhibited improved sensing properties compared to other forms. The humidity sensing properties of the sensors, including the sensitivity, hysteresis, response-recovery time, repeatability, and long-term stability, were evaluated at room temperature under exposure of various RH levels. Furthermore, the possible sensing mechanism of the ZnO/PVP-RGO sensors is also discussed in this paper. 

## 2. Experimental Section

### 2.1. Materials

ZnO nanopowders of a 50 ± 10 nm particle size and 99.8% metals basis were purchased from Aladdin. ZnO was dissolved in deionized water via magnetic stirring and sonication. ZnO aqueous suspension (20 mg/mL) was magnetically stirred well before each use to avoid precipitate, and 1 mg/mL PVP-RGO aqueous suspension and 5 mg/mL PVP aqueous solution were supplied by Xfnano Materials Tech. Co. Ltd. (Nanjing, China). The PVP/RGO mass ratio in the PVP-RGO aqueous suspension was 5:1. All of the above chemicals of analytical grade were used as received without any further purification.

### 2.2. Preparation of Sensors

The humidity sensors used in this study were all prepared on Corning eagle XG glass substrates with a 500 µm thickness. The aluminum interdigitated electrodes (IDEs) were defined by lithography and the electron beam evaporation process, which were composed of two main 100 µm wide tracks with 10 teeth. The width of teeth and the distance between them were all 50 µm and the outline dimension of the IDEs pattern was 1 × 1 mm. Before the drop-coating of sensing materials, the IDEs were wet-cleaned with acetone, ethanol, and deionized water, respectively, for 5 min. Finally, the surface of the IDEs was dried with nitrogen gas [22]. 

The typical procedure to prepare ZnO/PVP-RGO sensors is described as follows: 0.7 μL of ZnO aqueous suspension was dropped onto the surface of IDEs using a 10 μL pipette, followed by drying on a hot plate in the presence of air at 60 °C for 1 h. Then, 1.0 μL of PVP-RGO aqueous suspension was separated into two 0.5 μL drops, each of which were sequentially dropped onto the surface of the ZnO-coated IDEs and dried on a hot plate in the presence of air for 1 h at 60 °C. For comparison, similar procedures were used to fabricate PVP-RGO/ZnO, PVP-RGO, ZnO, and ZnO/PVP sensors.

### 2.3. Material Characterization and Humidity Sensing Measurements

**Figure 1:**
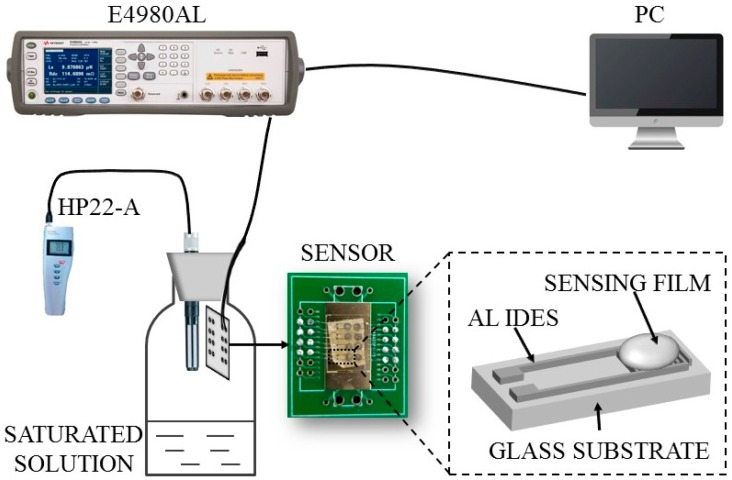
Schematic of the humidity measurement system and of the constructed humidity sensors.

The surface morphologies of prepared sensing films were observed by scanning electron microscopy (SEM, GeminiSEM500, Carl Zeiss, Oberkochen, Germany), and these sensing films were structurally characterized through X-ray diffractometer (XRD, D8-Advance, Bruker, Karlsruhe, Germany). A schematic illustration of the humidity measurement system is given in Figure 1. Different RH environments were obtained using eight types of saturated salt solution (LiCl, CH_3_COOK, MgCl_2_, K_2_CO_3_, NaBr, NaCl, KCl, and K_2_SO_4_) in a closed glass bottle. A commercial hygrometer (Rotronic HP22-A, ±0.8% RH) was placed inside the closed glass bottle for RH calibration, and all experimental measurements were carried out after the film was balanced with water molecules. The humidity sensing properties of the sensors were investigated by recording their capacitance responses to humidity at room temperature (~22 °C) using an LCR Meter (Keysight E4980AL, Santa Rosa, CA, USA). The applied AC and DC voltage had amplitudes of 100 mV and 0 V respectively, and the AC frequency was 100 Hz. The transient response times were obtained by the real-time monitoring of the sensors’ capacitance responses when they were quickly transferred between different humidity environments (LiCl and K_2_SO_4_).

## 3. Results and Discussion

### 3.1. Microstructure and Morphological Characterization

**Figure 2:**
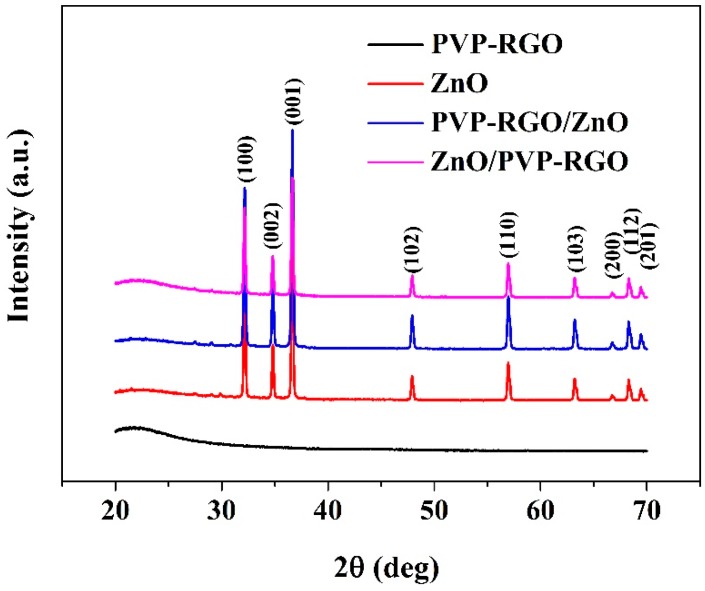
Typical XRD images of polyvinyl pyrrolidone–reduced graphene oxide (PVP-RGO), zinc oxide (ZnO), PVP-RGO/ZnO and ZnO/PVP-RGO films.

**Figure 3:**
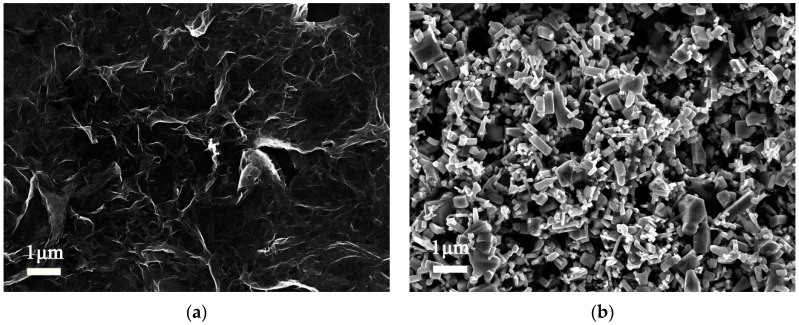

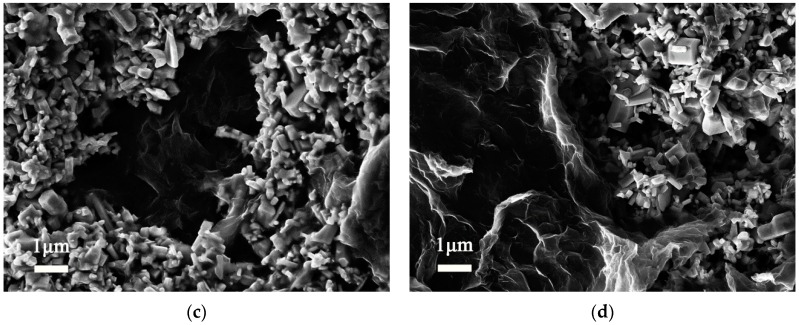
Typical SEM images of (**a**) PVP-RGO, (**b**) ZnO, (**c**) PVP-RGO/ZnO, and (**d**) ZnO/PVP-RGO films.

Figure 2 shows XRD patterns of PVP-RGO, ZnO, PVP-RGO/ZnO, and ZnO/PVP-RGO films. In ZnO, PVP-RGO/ZnO, and ZnO/PVP-RGO curves, all diffraction peaks can be indexed to the hexagonal wurtzite structure of ZnO. The intensity of the (101) peak is stronger than the (100) and (002) peaks in all of these samples [37,38]. Moreover, the broad peak from 20 to 33° in the curve of PVP-RGO reveals the presence of graphene sheets in the nanocomposities [39]. The typical SEM images of as-prepared PVP-RGO, ZnO, PVP-RGO/ZnO, and ZnO/PVP-RGO films are shown in Figure 3a–d, respectively. The PVP-RGO film in Figure 3a has obvious wrinkles and exhibits a continuous distribution. However, the ZnO nanopowders in Figure 3b are scattered throughout the substrate, forming a network of pores. These pores are expected to provide water moisture adsorption sites [24]. Each ZnO grain is a polyhedron with facets, edges, and vertices, indicating their polycrystalline structures [20]. Pure ZnO nanopowders with lengths in the range of approximately 0.1–1 μm and diameters in the range of 50–500 nm are observed. The length of most ZnO nanopowders is larger than 500 nm. In the case of PVP-RGO/ZnO film, part of the PVP-RGO film is covered by ZnO nanopowders and the others are exposed to air (see Figure 3c). In the case of the ZnO/PVP-RGO films, the PVP-RGO nanocomposites scattered throughout the ZnO substrate form a network structure. This connected multilayer structure contributes to the electron transfer between ZnO nanopowders and PVP-RGO nanocomposites, and it increases the surface area of the sensing film to improve the sensor’s sensitivity (see Figure 3d) [28]. 

### 3.2. Humidity Sensing Properties

**Figure 4:**
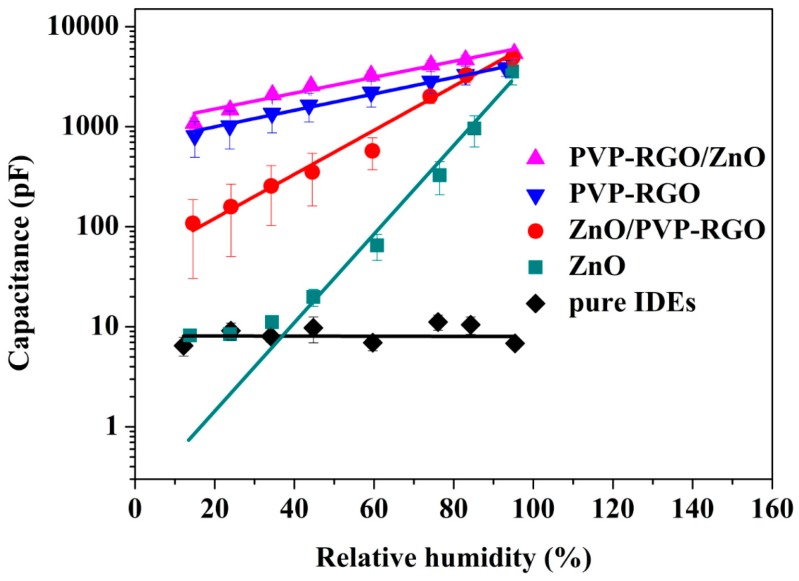
Variation in the capacitance with RH changes in ZnO, PVP-RGO, ZnO/PVP-RGO, and PVP-RGO/ZnO sensors and pure IDEs. The data points and error bars represent the average values and the standard deviations, respectively, from 11 separate sensors of the same material.

The humidity responses of ZnO, PVP-RGO, ZnO/PVP-RGO, and PVP-RGO/ZnO sensors and pure IDEs were measured for comparison. The response curves of all sensors and IDEs are as shown in Figure 4. As we can see, the pure IDEs were insensitive to humidity, while the measured capacitances, for all sensors, increased monotonically with the increase of RH and, except for the ZnO sensors at low RH (<40%), showed nearly linear responses in the logarithmic scale. Hence, the sensitivity was defined as the ratio of the logarithmic capacitance difference to the RH change (Δlog_10_C/ΔRH). Obviously, the sensitivity of the ZnO/PVP-RGO (~0.022) sensors was about three times larger than that of PVP-RGO (~0.008) and PVP-RGO/ZnO (~0.008) sensors in the RH range 15–95%. ZnO sensors exhibited the highest sensitivity (~0.044) at middle and high RH (>40%), while it was insensitive at low RH. One possible explanation for this is that the size of the ZnO nanopowders was relatively large, which meant that the ZnO film had a low surface area and exhibited a low affinity to external water molecules at a low RH range [40]. However, the PVP-RGO film had certain hydrophilic functional groups and exhibited a relatively high affinity for external water molecules; thus, PVP-RGO, PVP-RGO, and ZnO/PVP-RGO sensors showed better sensitivity at low RH. Overall, considering the high sensitivity and the good linearity, the ZnO/PVP-RGO sensors exhibited the best sensing performance, which mainly came from its special surface morphology and microstructures.

**Figure 5:**
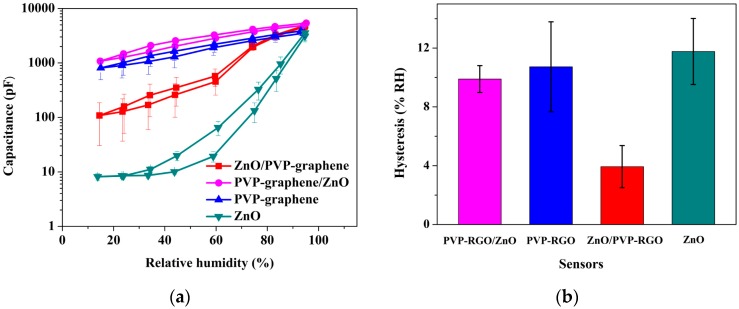
(**a**) The hysteresis characteristics of ZnO, PVP-RGO, ZnO/PVP-RGO, and PVP-RGO/ZnO sensors under different RHs. The solid and the dashed lines represent the desorption and adsorption processes, respectively. (**b**) Hysteresis statistics of ZnO, PVP-RGO, ZnO/PVP-RGO, and PVP-RGO/ZnO sensors.

It is well known that the humidity hysteresis in the absorption/desorption process will cause uncertainty when determining the RH value. Therefore, humidity hysteresis had to be as small as possible for practical use. Here, the hysteresis characteristics of ZnO, PVP-RGO, ZnO/PVP-RGO, and PVP-RGO/ZnO sensors are depicted in Figure 5a, and Figure 5b shows their statistical analyses. It is worth mentioning that the ZnO/PVP-RGO sensors had much smaller hysteresis (~3.9% RH) than the sensors based on PVP-RGO/ZnO (~9.9% RH), PVP-RGO (~10.7% RH), and ZnO (~11.8% RH). In our experiment, only the prepared ZnO/PVP-RGO sensors showed an acceptable hysteresis value less than 5% RH. A narrow hysteresis loop of ZnO/PVP-RGO sensors indicates that fast equilibrium can be achieved between the adsorption and desorption processes in the ZnO/PVP-RGO films.

**Figure 6:**
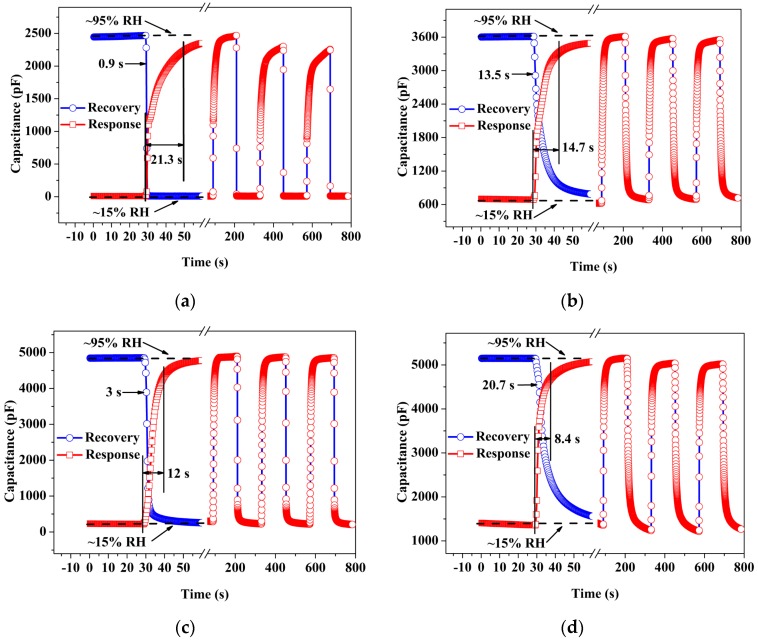
The response-recovery behavior and repeatability of (**a**) ZnO, (**b**) PVP-RGO, (**c**) ZnO/PVP-RGO, and (**d**) PVP-RGO/ZnO sensors when the relative humidity changed rapidly between 15% and 95% RH at room temperature.

Furthermore, the response–recovery behavior and repeatability are two important characteristics with which to evaluate the performance of the humidity sensors [19]. Figure 6a–d shows the typical response–recovery behavior and repeatability of ZnO, PVP-RGO, ZnO/PVP-RGO, and PVP-RGO/ZnO sensors for the same conditions. The details of the rise and fall of the sensors to humidity changes are all as given in Figure 6, where the time between each data point is 0.3 s. We defined the time it took to reach 90% and 10% of the total capacitance as the response time (t_90%_) in an RH-increasing process and the recovery time (t_10%_) in an RH-decreasing process, respectively. The response times of ZnO, PVP-RGO, ZnO/PVP-RGO, and PVP-RGO/ZnO sensor were 21.3 s, 14.7 s, 12.0 s, and 8.4 s, respectively. The recovery times of ZnO, PVP-RGO, ZnO/PVP-RGO, and PVP-RGO/ZnO sensors were 0.9 s, 13.5 s, 3.0 s, and 20.7 s, respectively. The repeatability characteristic was repeatedly measured for three exposure/recovery cycles by switching between 15% and 95% RH. All sensors exhibited a clear response–recovery behavior, and, for the ZnO sensor, the magnitude of the change in capacitance was larger in the first adsorption–desorption cycle compared to later repeated cycles due its slower adsorption rate [18]. In summary, the ZnO/PVP-RGO sensor exhibited the best response–recovery behavior and sensing repeatability, which enabled the detection and monitoring of water vapor in real time. The sensing properties for the ZnO/PVP-RGO sensors are comparable to those of capacitive-type humidity sensors in previous works, which are presented in Table 1. 

**Figure 7:**
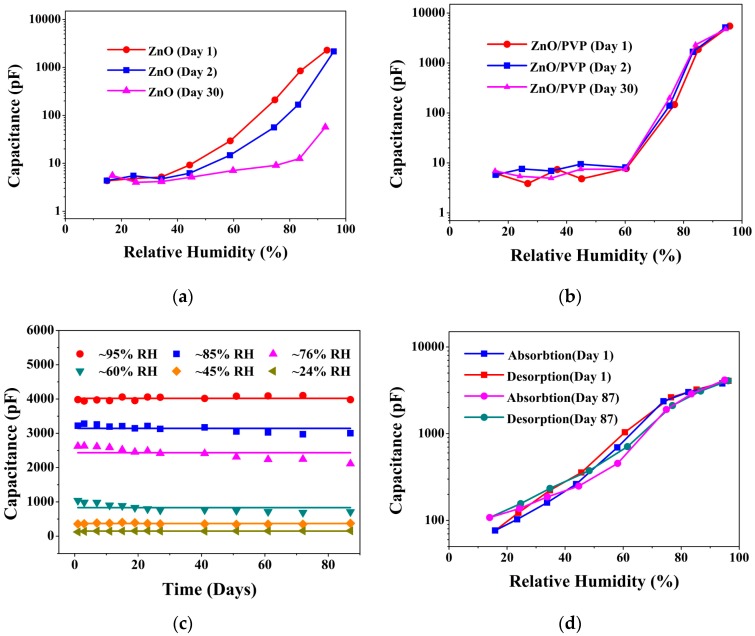
Capacitance vs. RH for (**a**) ZnO and (**b**) ZnO/PVP sensors in one month. (**c**) Stability of the ZnO/PVP-RGO sensor under various RH conditions. (**d**) Capacitance vs. RH for ZnO/PVP-RGO sensor, showing good stability and little hysteresis over 87 days.

To understand the effect of PVP on the ZnO/PVP-RGO sensor’s performance, how the capacitance response of ZnO and ZnO/PVP sensors changed over days is shown in Figure 7a,b, respectively. The curves clearly show that the capacitance response of ZnO/PVP was virtually unchanged as the measurement time increased, while the capacitance response of ZnO diminished gradually. That is to say, PVP was able to improve the sensor’s stability. This might be because the addition of PVP leads to stability in the surface of the ZnO nanopowders. After exposure to ambient for 87 days at room temperature, the ZnO/PVP-RGO sensor was regularly monitored to test its stability. The time evolution of the capacitance value for the ZnO/PVP-RGO sensor measured at different RH levels is as shown in Figure 7c. The device showed satisfactory long-term stability for humidity detection. Figure 7d shows the long-term hysteresis stability in the ZnO/PVP-RGO sensor, which shows good stability and little hysteresis over the 87 day period.

### 3.3. Discussion of the Sensing Mechanism

Traditionally, the basic mechanism for capacitive-type humidity sensors is electrical permittivity that is sensitive to humidity variation, and the electrical response that occurs in a humidity sensor is linked to the water adsorption–desorption process that takes place on the exposed surface of the sensing material [30]. Water condensation tends to occur within the porous structure of the sensing materials. Since water has very high permittivity at room temperature (~80) compared to air, the effective dielectric constant of the whole sensing material will change in response to water adsorption or desorption [43]. 

The above experimental results demonstrate that the fabricated ZnO/PVP-RGO sensor is very sensitive to RH changes, highlighting that ZnO/PVP-RGO is an excellent candidate material for the construction of humidity sensors. The reason for ZnO/PVP-RGO sensor’s excellent sensing performance can be attributed to four aspects. First, ZnO/PVP-RGO has a super surface-to-volume ratio, which can increase the sensor’s sensitivity. Second, the PVP-RGO coating decreases the distance between ZnO nanopowders. The interstices of nanostructures provide an effective pathway for vapor transportation and are helpful in the rapid adsorption and desorption of water molecules. Furthermore, RGO has low resistivity and high carrier mobility, serving as an anchor with which to promote electron transfer in the metal oxide nanoparticles, which plays a role in modification of ZnO [33]. Third, the heterojunction may be created at the interface of the two nanomaterials and contributes to the improvement in humidity sensing [28]. Fourth, the intrinsic chemical stability of PVP can enhance the sensor’s stability and repeatability [42].

## 4. Conclusions

This paper demonstrated a facile fabrication of humidity sensor based on a ZnO/PVP-RGO multilayer structure and its humidity sensing properties were investigated at room temperature. Moreover, the humidity properties of different sensing films were investigated for comparison. Significant improvements in both sensitivity and linearity were achieved for the ZnO/PVP-RGO sensors compared with the PVP-RGO/ZnO, PVP-RGO, and ZnO counterparts. Additionally, the ZnO/PVP-RGO sensor exhibited small hysteresis (~3.9% RH), fast response and recovery times over a full humidity range measurement (t_90%_ of 12.0 s and a t_10%_ of 3.0 s during adsorption and desorption processes, respectively), and long-term stability. Finally, the possible humidity sensing mechanism of the ZnO/PVP-RGO sensor was also discussed. This study indicated that ZnO/PVP-RGO may be a promising candidate material for the fabrication of high-performance humidity sensors.

## Figures and Tables

**Table 1:** Comparison in performance towards previous work.

Sensing Material	Fabrication Method	Measurement Range	Response/Recovery Time	Hysteresis	Reference
ZnO/PVP-RGO	Drop-coating	15–95% RH	12 s/3 s	~3.9% RH	This work
ZnO	Sol-gel method	55–90% RH	250 s/-	-	[11]
GO	Drop-casting	23–86% RH	10.5 s/41 s	-	[16]
PEPC+NiPC+Cu_2_O	Spin-coating	40–97% RH	13 s/15 s	~12% RH	[41]
ZnO/Si	Sol-gel method	11.3–97.7% RH	26 s/7 s	~0.79% RH	[42]
